# Supplementary material for: Isolation of Clostridioides difficile from a Large Animal Veterinary Teaching Hospital Environment
Source: Animals (Basel). 2025 Sep 15;15(18):2703. doi: 10.3390/ani15182703 (PMC12466365; doi:10.3390/ani15182703)
Supplement: Supplementary file 1 [file animals-15-02703-s001.zip › animals-3840555-supplementary.pdf]

Supplementary Table S1. Sequence Type and Assigned Clade for *C. difficile* RefSeq Genomes[1] (n=114) and Guelph Environmental Isolates (n= 14).

Genome	ST [2]	CLADE [3]	MLST alleles						
GCF_001577795.1	2	1	adk(1)	atpA(1)	dxr(2)	glyA(1)	recA(5)	sodA(3)	tpi(1)
GCF_002812625.1	2	1	adk(1)	atpA(1)	dxr(2)	glyA(1)	recA(5)	sodA(3)	tpi(1)
GCF_018884745.1	2	1	adk(1)	atpA(1)	dxr(2)	glyA(1)	recA(5)	sodA(3)	tpi(1)
GCF_018885065.1	2	1	adk(1)	atpA(1)	dxr(2)	glyA(1)	recA(5)	sodA(3)	tpi(1)
GCF_024399435.1	2	1	adk(1)	atpA(1)	dxr(2)	glyA(1)	recA(5)	sodA(3)	tpi(1)
GCF_001077535.1	3	1	adk(1)	atpA(1)	dxr(2)	glyA(1)	recA(1)	sodA(1)	tpi(1)
GCF_002073735.2	3	1	adk(1)	atpA(1)	dxr(2)	glyA(1)	recA(1)	sodA(1)	tpi(1)
GCF_003482345.1	3	1	adk(1)	atpA(1)	dxr(2)	glyA(1)	recA(1)	sodA(1)	tpi(1)
GCF_018884705.1	3	1	adk(1)	atpA(1)	dxr(2)	glyA(1)	recA(1)	sodA(1)	tpi(1)
GCF_002812645.1	8	1	adk(1)	atpA(1)	dxr(2)	glyA(6)	recA(1)	sodA(5)	tpi(1)
GCF_018885085.1	8	1	adk(1)	atpA(1)	dxr(2)	glyA(6)	recA(1)	sodA(5)	tpi(1)
GCF_018884845.1	10	1	adk(2)	atpA(1)	dxr(2)	glyA(1)	recA(1)	sodA(3)	tpi(1)
GCF_018884905.1	10	1	adk(2)	atpA(1)	dxr(2)	glyA(1)	recA(1)	sodA(3)	tpi(1)
GCF_018884765.1	14	1	adk(1)	atpA(1)	dxr(2)	glyA(1)	recA(5)	sodA(5)	tpi(3)
Guelph19	14	1	adk(1)	atpA(1)	dxr(2)	glyA(1)	recA(5)	sodA(5)	tpi(3)
2117	14	1	adk(1)	atpA(1)	dxr(2)	glyA(1)	recA(5)	sodA(5)	tpi(3)
GCF_003481905.1	15	1	adk(1)	atpA(1)	dxr(6)	glyA(1)	recA(8)	sodA(5)	tpi(1)
GCF_025135955.1	15	1	adk(1)	atpA(1)	dxr(6)	glyA(1)	recA(8)	sodA(5)	tpi(1)
Guelph88	15	1	adk(1)	atpA(1)	dxr(6)	glyA(1)	recA(8)	sodA(5)	tpi(1)
Guelph9	15	1	adk(1)	atpA(1)	dxr(6)	glyA(1)	recA(8)	sodA(5)	tpi(1)
Guelph92	15	1	adk(1)	atpA(1)	dxr(6)	glyA(1)	recA(8)	sodA(5)	tpi(1)
Guelph12	26	1	adk(1)	atpA(1)	dxr(6)	glyA(1)	recA(4)	sodA(3)	tpi(4)
Guelph185	26	1	adk(1)	atpA(1)	dxr(6)	glyA(1)	recA(4)	sodA(3)	tpi(4)
Guelph8	26	1	adk(1)	atpA(1)	dxr(6)	glyA(1)	recA(4)	sodA(3)	tpi(4)
GCF_025136135.1	29	1	adk(1)	atpA(1)	dxr(2)	glyA(16)	recA(1)	sodA(3)	tpi(1)
GCF_008245165.1	34	1	adk(1)	atpA(5)	dxr(7)	glyA(1)	recA(1)	sodA(3)	tpi(1)
GCF_018885105.1	34	1	adk(1)	atpA(5)	dxr(7)	glyA(1)	recA(1)	sodA(3)	tpi(1)
GCF_002007885.1	42	1	adk(1)	atpA(1)	dxr(2)	glyA(1)	recA(1)	sodA(7)	tpi(1)
GCF_002234355.1	42	1	adk(1)	atpA(1)	dxr(2)	glyA(1)	recA(1)	sodA(7)	tpi(1)
GCF_002812605.1	42	1	adk(1)	atpA(1)	dxr(2)	glyA(1)	recA(1)	sodA(7)	tpi(1)
GCF_018884825.1	42	1	adk(1)	atpA(1)	dxr(2)	glyA(1)	recA(1)	sodA(7)	tpi(1)
GCF_018884865.1	43	1	adk(1)	atpA(7)	dxr(6)	glyA(1)	recA(1)	sodA(5)	tpi(6)
GCF_018884925.1	43	1	adk(1)	atpA(7)	dxr(6)	glyA(1)	recA(1)	sodA(5)	tpi(6)
GCF_018884945.1	43	1	adk(1)	atpA(7)	dxr(6)	glyA(1)	recA(1)	sodA(5)	tpi(6)
Isolate1 [4]	43	1	adk(1)	atpA(7)	dxr(6)	glyA(1)	recA(1)	sodA(5)	tpi(6)
Isolate2 [4]	43	1	adk(1)	atpA(7)	dxr(6)	glyA(1)	recA(1)	sodA(5)	tpi(6)
GCF_015238635.1	46	1	adk(4)	atpA(1)	dxr(6)	glyA(1)	recA(1)	sodA(10)	tpi(1)
GCF_003482065.1	48	1	adk(1)	atpA(1)	dxr(2)	glyA(1)	recA(1)	sodA(5)	tpi(1)
GCF_014236775.1	49	1	adk(1)	atpA(1)	dxr(2)	glyA(1)	recA(5)	sodA(3)	tpi(3)

GCF_020097215.1	49	1	adk(1)	atpA(1)	dxr(2)	glyA(1)	recA(5)	sodA(3)	tpi(3)
GCF_018884665.1	53	1	adk(1)	atpA(2)	dxr(2)	glyA(1)	recA(1)	sodA(5)	tpi(1)
GCF_000009205.2	54	1	adk(1)	atpA(4)	dxr(7)	glyA(1)	recA(1)	sodA(3)	tpi(3)
GCF_000932055.2	54	1	adk(1)	atpA(4)	dxr(7)	glyA(1)	recA(1)	sodA(3)	tpi(3)
GCF_000953275.1	54	1	adk(1)	atpA(4)	dxr(7)	glyA(1)	recA(1)	sodA(3)	tpi(3)
GCF_002080065.1	54	1	adk(1)	atpA(4)	dxr(7)	glyA(1)	recA(1)	sodA(3)	tpi(3)
GCF_003313565.1	54	1	adk(1)	atpA(4)	dxr(7)	glyA(1)	recA(1)	sodA(3)	tpi(3)
GCF_018884965.1	54	1	adk(1)	atpA(4)	dxr(7)	glyA(1)	recA(1)	sodA(3)	tpi(3)
GCF_022695655.1	54	1	adk(1)	atpA(4)	dxr(7)	glyA(1)	recA(1)	sodA(3)	tpi(3)
Guelph51	54	1	adk(1)	atpA(4)	dxr(7)	glyA(1)	recA(1)	sodA(3)	tpi(3)
Guelph54	54	1	adk(1)	atpA(4)	dxr(7)	glyA(1)	recA(1)	sodA(3)	tpi(3)
Guelph82	54	1	adk(1)	atpA(4)	dxr(7)	glyA(1)	recA(1)	sodA(3)	tpi(3)
Guelph94	54	1	adk(1)	atpA(4)	dxr(7)	glyA(1)	recA(1)	sodA(3)	tpi(3)
GCF_003482325.1	83	1	adk(1)	atpA(1)	dxr(6)	glyA(1)	recA(1)	sodA(3)	tpi(1)
GCF_003481965.1	103	1	adk(1)	atpA(1)	dxr(2)	glyA(15)	recA(8)	sodA(5)	tpi(3)
GCF_018884605.1	110	1	adk(1)	atpA(1)	dxr(2)	glyA(1)	recA(13)	sodA(3)	tpi(1)
GCF_018884885.1	185	1	adk(2)	atpA(1)	dxr(16)	glyA(1)	recA(5)	sodA(5)	tpi(1)
GCF_009867095.1	203	1	adk(1)	atpA(22)	dxr(2)	glyA(1)	recA(22)	sodA(1)	tpi(1)
GCF_003490105.1	701	1	adk(1)	atpA(1)	dxr(2)	glyA(1)	recA(1)	sodA(12)	tpi(6)
GCF_001984465.1	UNK [5]	1	adk(1)	atpA(1)	dxr(2)	glyA(45?)	recA(5)	sodA(3)	tpi(1)
GCF_000085225.1	1	2	adk(1)	atpA(1)	dxr(1)	glyA(10)	recA(1)	sodA(3)	tpi(5)
GCF_000210455.1	1	2	adk(1)	atpA(1)	dxr(1)	glyA(10)	recA(1)	sodA(3)	tpi(5)
GCF_000211235.1	1	2	adk(1)	atpA(1)	dxr(1)	glyA(10)	recA(1)	sodA(3)	tpi(5)
GCF_002812585.1	1	2	adk(1)	atpA(1)	dxr(1)	glyA(10)	recA(1)	sodA(3)	tpi(5)
GCF_002945415.1	1	2	adk(1)	atpA(1)	dxr(1)	glyA(10)	recA(1)	sodA(3)	tpi(5)
GCF_002945515.1	1	2	adk(1)	atpA(1)	dxr(1)	glyA(10)	recA(1)	sodA(3)	tpi(5)
GCF_002945665.1	1	2	adk(1)	atpA(1)	dxr(1)	glyA(10)	recA(1)	sodA(3)	tpi(5)
GCF_002945755.1	1	2	adk(1)	atpA(1)	dxr(1)	glyA(10)	recA(1)	sodA(3)	tpi(5)
GCF_002945855.1	1	2	adk(1)	atpA(1)	dxr(1)	glyA(10)	recA(1)	sodA(3)	tpi(5)
GCF_002945945.1	1	2	adk(1)	atpA(1)	dxr(1)	glyA(10)	recA(1)	sodA(3)	tpi(5)
GCF_002946035.1	1	2	adk(1)	atpA(1)	dxr(1)	glyA(10)	recA(1)	sodA(3)	tpi(5)
GCF_002946135.1	1	2	adk(1)	atpA(1)	dxr(1)	glyA(10)	recA(1)	sodA(3)	tpi(5)
GCF_002946195.1	1	2	adk(1)	atpA(1)	dxr(1)	glyA(10)	recA(1)	sodA(3)	tpi(5)
GCF_003313545.1	1	2	adk(1)	atpA(1)	dxr(1)	glyA(10)	recA(1)	sodA(3)	tpi(5)
GCF_003313585.1	1	2	adk(1)	atpA(1)	dxr(1)	glyA(10)	recA(1)	sodA(3)	tpi(5)
GCF_003482035.1	1	2	adk(1)	atpA(1)	dxr(1)	glyA(10)	recA(1)	sodA(3)	tpi(5)
GCF_009730495.1	1	2	adk(1)	atpA(1)	dxr(1)	glyA(10)	recA(1)	sodA(3)	tpi(5)
GCF_015732555.1	1	2	adk(1)	atpA(1)	dxr(1)	glyA(10)	recA(1)	sodA(3)	tpi(5)
GCF_018255775.1	1	2	adk(1)	atpA(1)	dxr(1)	glyA(10)	recA(1)	sodA(3)	tpi(5)
GCF_021378415.1	1	2	adk(1)	atpA(1)	dxr(1)	glyA(10)	recA(1)	sodA(3)	tpi(5)
GCF_025758145.1	1	2	adk(1)	atpA(1)	dxr(1)	glyA(10)	recA(1)	sodA(3)	tpi(5)
GCF_025758165.1	1	2	adk(1)	atpA(1)	dxr(1)	glyA(10)	recA(1)	sodA(3)	tpi(5)
GCF_025758185.1	1	2	adk(1)	atpA(1)	dxr(1)	glyA(10)	recA(1)	sodA(3)	tpi(5)

GCF_025758205.1	1	2	adk(1)	atpA(1)	dxr(1)	glyA(10)	recA(1)	sodA(3)	tpi(5)
GCF_025758225.1	1	2	adk(1)	atpA(1)	dxr(1)	glyA(10)	recA(1)	sodA(3)	tpi(5)
GCF_025758245.1	1	2	adk(1)	atpA(1)	dxr(1)	glyA(10)	recA(1)	sodA(3)	tpi(5)
GCF_018885045.1	188	2	adk(1)	atpA(1)	dxr(9)	glyA(9)	recA(14)	sodA(5)	tpi(2)
GCF_003456975.1	5	3	adk(1)	atpA(6)	dxr(4)	glyA(7)	recA(2)	sodA(8)	tpi(7)
GCF_003457015.1	5	3	adk(1)	atpA(6)	dxr(4)	glyA(7)	recA(2)	sodA(8)	tpi(7)
GCF_003457035.1	5	3	adk(1)	atpA(6)	dxr(4)	glyA(7)	recA(2)	sodA(8)	tpi(7)
GCF_003095675.1	37	4	adk(3)	atpA(7)	dxr(3)	glyA(8)	recA(6)	sodA(9)	tpi(11)
GCF_003095695.1	37	4	adk(3)	atpA(7)	dxr(3)	glyA(8)	recA(6)	sodA(9)	tpi(11)
GCF_003482255.1	37	4	adk(3)	atpA(7)	dxr(3)	glyA(8)	recA(6)	sodA(9)	tpi(11)
GCF_019095765.1	37	4	adk(3)	atpA(7)	dxr(3)	glyA(8)	recA(6)	sodA(9)	tpi(11)
GCF_024972115.1	37	4	adk(3)	atpA(7)	dxr(3)	glyA(8)	recA(6)	sodA(9)	tpi(11)
GCF_024972275.1	37	4	adk(3)	atpA(7)	dxr(3)	glyA(8)	recA(6)	sodA(9)	tpi(11)
GCF_003482225.1	38	4	adk(3)	atpA(7)	dxr(10)	glyA(8)	recA(6)	sodA(2)	tpi(9)
GCF_003482305.1	39	4	adk(3)	atpA(7)	dxr(10)	glyA(8)	recA(7)	sodA(2)	tpi(10)
GCF_022845595.1	81	4	adk(3)	atpA(1)	dxr(3)	glyA(8)	recA(6)	sodA(9)	tpi(11)
GCF_024972415.1	81	4	adk(3)	atpA(1)	dxr(3)	glyA(8)	recA(6)	sodA(9)	tpi(11)
GCF_024972555.1	81	4	adk(3)	atpA(1)	dxr(3)	glyA(8)	recA(6)	sodA(9)	tpi(11)
GCF_000210415.1	86	4	adk(3)	atpA(7)	dxr(3)	glyA(8)	recA(6)	sodA(19)	tpi(11)
GCF_003482125.1	109	4	adk(3)	atpA(12)	dxr(10)	glyA(18)	recA(6)	sodA(18)	tpi(15)
GCF_000210435.1	11	5	adk(5)	atpA(8)	dxr(5)	glyA(11)	recA(9)	sodA(11)	tpi(8)
GCF_002946515.2	11	5	adk(5)	atpA(8)	dxr(5)	glyA(11)	recA(9)	sodA(11)	tpi(8)
GCF_002946535.2	11	5	adk(5)	atpA(8)	dxr(5)	glyA(11)	recA(9)	sodA(11)	tpi(8)
GCF_002946555.2	11	5	adk(5)	atpA(8)	dxr(5)	glyA(11)	recA(9)	sodA(11)	tpi(8)
GCF_003482165.1	11	5	adk(5)	atpA(8)	dxr(5)	glyA(11)	recA(9)	sodA(11)	tpi(8)
GCF_003482365.1	11	5	adk(5)	atpA(8)	dxr(5)	glyA(11)	recA(9)	sodA(11)	tpi(8)
GCF_003697205.1	11	5	adk(5)	atpA(8)	dxr(5)	glyA(11)	recA(9)	sodA(11)	tpi(8)
GCF_003697225.1	11	5	adk(5)	atpA(8)	dxr(5)	glyA(11)	recA(9)	sodA(11)	tpi(8)
GCF_009362915.1	11	5	adk(5)	atpA(8)	dxr(5)	glyA(11)	recA(9)	sodA(11)	tpi(8)
GCF_015732535.1	11	5	adk(5)	atpA(8)	dxr(5)	glyA(11)	recA(9)	sodA(11)	tpi(8)
GCF_016766955.1	11	5	adk(5)	atpA(8)	dxr(5)	glyA(11)	recA(9)	sodA(11)	tpi(8)
GCF_016766975.1	11	5	adk(5)	atpA(8)	dxr(5)	glyA(11)	recA(9)	sodA(11)	tpi(8)
GCF_016766995.1	11	5	adk(5)	atpA(8)	dxr(5)	glyA(11)	recA(9)	sodA(11)	tpi(8)
GCF_016767015.1	11	5	adk(5)	atpA(8)	dxr(5)	glyA(11)	recA(9)	sodA(11)	tpi(8)
GCF_016767035.1	11	5	adk(5)	atpA(8)	dxr(5)	glyA(11)	recA(9)	sodA(11)	tpi(8)
GCF_016767055.1	11	5	adk(5)	atpA(8)	dxr(5)	glyA(11)	recA(9)	sodA(11)	tpi(8)
GCF_016767095.1	11	5	adk(5)	atpA(8)	dxr(5)	glyA(11)	recA(9)	sodA(11)	tpi(8)
GCF_016767115.1	11	5	adk(5)	atpA(8)	dxr(5)	glyA(11)	recA(9)	sodA(11)	tpi(8)
GCF_016767135.1	11	5	adk(5)	atpA(8)	dxr(5)	glyA(11)	recA(9)	sodA(11)	tpi(8)
GCF_017592625.2	11	5	adk(5)	atpA(8)	dxr(5)	glyA(11)	recA(9)	sodA(11)	tpi(8)
GCF_018603395.1	11	5	adk(5)	atpA(8)	dxr(5)	glyA(11)	recA(9)	sodA(11)	tpi(8)
GCF_018603455.1	11	5	adk(5)	atpA(8)	dxr(5)	glyA(11)	recA(9)	sodA(11)	tpi(8)
GCF_018603475.1	11	5	adk(5)	atpA(8)	dxr(5)	glyA(11)	recA(9)	sodA(11)	tpi(8)

GCF_018884725.1	11	5	adk(5)	atpA(8)	dxr(5)	glyA(11)	recA(9)	sodA(11)	tpi(8)
GCF_019931025.1	11	5	adk(5)	atpA(8)	dxr(5)	glyA(11)	recA(9)	sodA(11)	tpi(8)
LAR19 [4]	11	5	adk(5)	atpA(8)	dxr(5)	glyA(11)	recA(9)	sodA(11)	tpi(8)
LAR25 [4]	11	5	adk(5)	atpA(8)	dxr(5)	glyA(11)	recA(9)	sodA(11)	tpi(8)
GCF_003697245.1	UNK[6]	5	adk(55)	atpA(8)	dxr(5)	glyA(11)	recA(9)	sodA(11)	tpi(8)

[1] https://www.ncbi.nlm.nih.gov/refseq/

[2] ST were determined from whole genome sequence files (contigs) using mlst software (Seemann T, **mlst** Github https://github.com/tseemann/mlst) based on the PubMLST website (https://pubmlst.org/) [20,21].

[3] Assignment of ST to Clades was based on Knight *et al*. eLife;10:e64325 (2021)* . All Clade. assignments were consistent with the core gene SNP tree (Supplementary Figure 1).

[4] Four equine infection isolates from the Guelph Lage Animal Hospital were included in the core gene SNP tree. Isolate 1 ad 2 are independent isolates belonging to ST43 (Clade 1). LAR19 and LAR25 are match mare and foal isolates belonging to ST11 (Clade 5).

[5] No Sequence Type match was present in the PubMLST.org database (https://pubmlst.org/organisms/clostridioides-difficile). The genomes were assigned to Clade 1 based on its position in the core gene SNP tree (Figure 1).

[6] No Sequence Type match was present in the PubMLST.org database (https://pubmlst.org/organisms/clostridioides-difficile). The genomes were assigned to Clade 5 based on its position in the core gene SNP tree (Supplemental Figure 1).

* Knight, D.R.; Imwattana, K; et al. Major genetic discontinuity and novel toxigenic species in *Clostridioides difficile* taxonomy. Elife. 2021, 10:e64325.

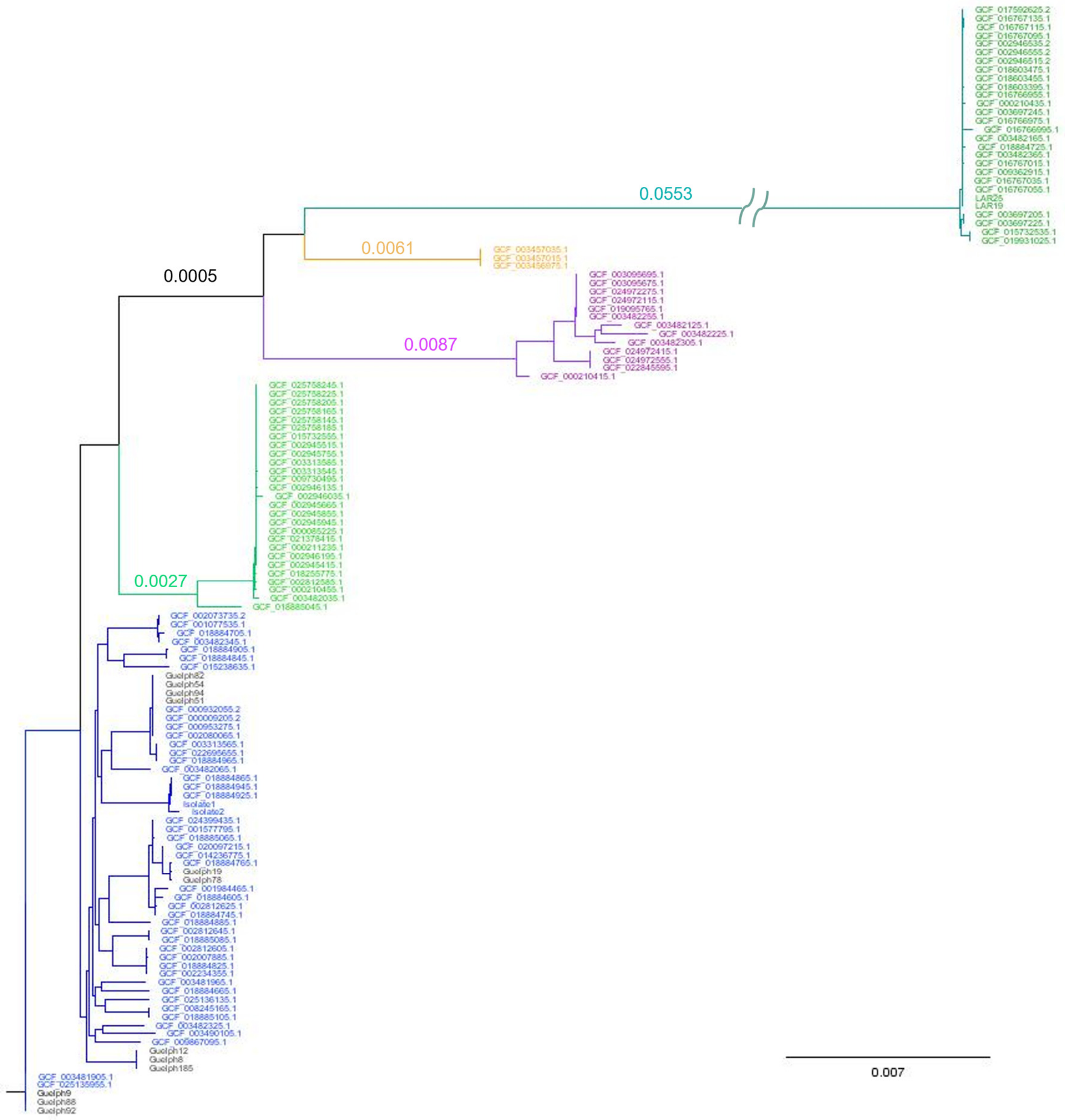

Supplementary Figure S1. *C. difficile* phylogenetic tree of environmental isolates with 114 reference genomes spanning clades 1-5 as in Figure 1 without Clades 2-5 collapsed.
